# The Effects of Reconditioning Exercises Following Prolonged Bed Rest on Lumbopelvic Muscle Volume and Accumulation of Paraspinal Muscle Fat

**DOI:** 10.3389/fphys.2022.862793

**Published:** 2022-06-14

**Authors:** Enrico De Martino, Julie Hides, James M. Elliott, Mark A. Hoggarth, Jochen Zange, Kirsty Lindsay, Dorothée Debuse, Andrew Winnard, David Beard, Jonathan A. Cook, Sauro E. Salomoni, Tobias Weber, Jonathan Scott, Paul W. Hodges, Nick Caplan

**Affiliations:** ^1^ Aerospace Medicine and Rehabilitation Laboratory, Faculty of Health and Life Sciences, Northumbria University, Newcastle upon Tyne, United Kingdom; ^2^ School of Health Sciences and Social Work, Griffith University, Brisbane, QLD, Australia; ^3^ Department of Physical Therapy and Human Movement Sciences, Feinberg School of Medicine, Northwestern University, Chicago, IL, United States; ^4^ Northern Sydney Local Health District, Faculty of Medicine and Health, The Kolling Institute Sydney, The University of Sydney, Sydney, NSW, Australia; ^5^ Department of Biomedical Engineering, McCormick School of Engineering, Northwestern University, Evanston, IL, United States; ^6^ German Aerospace Center, Institute of Aerospace Medicine, Cologne, Germany; ^7^ Nuffield Department of Orthopaedics, Rheumatology and Musculoskeletal Sciences, NIHR Oxford Biomedical Research Centre, University of Oxford, Oxford, United Kingdom; ^8^ Nuffield Department of Orthopaedics, Rheumatology and Musculoskeletal Sciences, Centre for Statistics in Medicine, University of Oxford, Oxford, United Kingdom; ^9^ NHMRC Centre for Clinical Research Excellence in Spinal Pain, Injury and Health, School of Health and Rehabilitation Sciences, The University of Queensland, Brisbane, QLD, Australia; ^10^ Space Medicine Team, European Astronaut Centre, Cologne, Germany; ^11^ KBR GmbH, Cologne, Germany

**Keywords:** space flight analogue, AGBRESA study, lumbar multifidus muscle, fatty infiltration, Dixon sequence, magnetic resonance imaging, reconditioning training

## Abstract

Reduced muscle size and accumulation of paraspinal muscle fat content (PFC) have been reported in lumbopelvic muscles after spaceflights and head-down tilt (HDT) bed rest. While some information is available regarding reconditioning programs on muscle atrophy recovery, the effects on the accumulation of PFC are unknown. Recently, a device (the Functional Re-adaptive Exercise Device—FRED) has been developed which aims to specifically recruit lumbopelvic muscles. This study aimed to investigate the effects of a standard reconditioning (SR) program and SR program supplemented by FRED (SR + FRED) on the recovery of the lumbopelvic muscles following 60-day HDT bed rest. Twenty-four healthy participants arrived at the facility for baseline data collection (BDC) before the bed rest period. They remained in the facility for 13-day post-HDT bed rest and were randomly allocated to one of two reconditioning programs: SR or SR + FRED. Muscle volumes of the lumbar multifidus (LM), lumbar erector spinae (LES), quadratus lumborum (QL), and psoas major (PM) muscles were measured from axial T1-weighted magnetic resonance imaging (MRI) at all lumbar intervertebral disc levels. PFC was determined using a chemical shift-based lipid/water Dixon sequence. Each lumbopelvic muscle was segmented into four equal quartiles (from medial to lateral). MRI of the lumbopelvic region was conducted at BDC, Day-59 of bed rest (HDT59), and Day-13 after reconditioning (R13). Comparing R13 with BDC, the volumes of the LM muscle at L4/L5 and L5/S1, LES at L1/L2, and QL at L3/L4 had not recovered (all—*p* < 0.05), and the PM muscle remained larger at L1/L2 (*p* = 0.001). Accumulation of PFC in the LM muscle at the L4/L5 and L5/S1 levels remained higher in the centro-medial regions at R13 than BDC (all—*p* < 0.05). There was no difference between the two reconditioning programs. A 2-week reconditioning program was insufficient to fully restore all volumes of lumbopelvic muscles and reverse the accumulation of PFC in the muscles measured to BDC values, particularly in the LM muscle at the lower lumbar levels. These findings suggest that more extended reconditioning programs or alternative exercises may be necessary to fully restore the size and properties of the lumbopelvic muscles after prolonged bed rest.

## Introduction

Decreased axial loading, such as experienced during spaceflight or prolonged bed rest, affects the active and passive elements of the lumbar spine. Adverse effects include lumbopelvic muscle atrophy (Hides et al., 2007, 2020), accumulation of paraspinal muscle fat (Burkhart et al., 2019), reduction in the lumbar lordosis (Belavý et al., 2008; Bailey et al., 2018), and alterations in the hydration status of intervertebral discs (IVD) (Kordi et al., 2015). Together, these adaptations are defined as “lumbar spine deconditioning,” a preclinical condition that may increase the risk of injuries to IVD and low back pain (LBP) (Belavý et al., 2016). Whilst in space, astronauts train daily to mitigate lumbar spine deconditioning and prevent IVD injuries, pain, and functional disability when re-exposed to gravity on Earth (Johnston et al., 2010). Furthermore, after returning from space, astronauts undertake 3 weeks of intense, progressive reconditioning to restore lumbar spine morphology and function (Petersen et al., 2016; Lambrecht et al., 2017). Reconditioning programs currently delivered after spaceflight follow many of the same principles as those used to rehabilitate patients with LBP (Hides et al., 2019).

Head-down tilt (HDT) bed rest studies provide an accelerated model of lumbar spine deconditioning (Hargens and Vico, 2016), which can be used to test novel rehabilitation procedures after periods of reduced axial loading with minimal confounding factors. Studies using magnetic resonance imaging (MRI) have demonstrated atrophy of the lumbar multifidus (LM), lumbar erector spinae (LES), and quadratus lumborum (QL) muscles and hypertrophy of the psoas major (PM) muscle after HDT bed rest (Hides et al., 2007; Belavý et al., 2010; De Martino et al., 2021b). Specific exercises focused on the recruitment and training of lumbopelvic muscles have been shown to reverse some of these adaptations (Hides et al., 2011). Magnetic resonance imaging can be used to quantify both muscle size and properties of muscle tissue, such as paraspinal muscle fat content (PFC) (Elliott et al., 2013), which has been shown to increase in the LM and LES muscles after 60-days of HDT bed rest (De Martino et al., 2021b) and spaceflight (Burkhart et al., 2019; McNamara et al., 2019). To date, no studies have investigated whether reconditioning programs after HDT bed rest can fully reverse the increased PFC in the lumbopelvic muscles. A detailed analysis of the lumbopelvic muscles at all lumbar vertebral levels is necessary to fully understand the effects of reconditioning programs on the recovery of PFC following HDT bed rest. This is particularly important considering that the spatial distribution of PFC in the LM and LES muscles depends on the muscle location and intervertebral level, where the most medial and lateral regions of the LM and LES at lower lumbar regions have proportionally greater PFC after 60 days of HDT bed rest (De Martino et al., 2021a). The spatial accumulation of fat in the LM and LES muscles also appears to be dependent on lumbar vertebral level [more significant accumulation at lower levels (Lee et al., 2017; Crawford et al., 2019)] as well as muscle location, with PFC being proportionally greater in the most medial regions in the elderly population (Crawford et al., 2019).

A new exercise device, called the Functional Re-adaptive Exercise Device (FRED), has been designed to recruit lumbopelvic muscles while performing a cyclical walking-type movement without external resistance within the device (Debuse et al., 2013). Due to the lack of external resistance to foot motion, as one foot moves downwards through the front of the movement cycle, the muscles in the user’s rear leg have to work eccentrically to maintain a smooth and controlled motion of the lower limbs (Debuse et al., 2013). The user aims to perform the movement of lower limbs with minimal variability in movement speed while the lumbar spine is held in a neutral position (Winnard et al., 2017b). Compared with walking, electromyography studies have provided evidence that the LM muscle is activated continuously throughout cycles on the FRED device (Caplan et al., 2014; Weber et al., 2017). It has been proposed that this movement pattern may have therapeutic benefits for people with LBP, and a proof-of-concept study demonstrated an increased size of the LM muscle after 6-week of training (Lindsay et al., 2020).

The first aim of the present study was to determine the effectiveness of a 2-week standard reconditioning (SR) program on the recovery of the volume and accumulation of PFC in the lumbopelvic muscles following 60-day HDT bed rest. The second aim was to investigate whether the application of FRED, in addition to the SR program (SR + FRED), could enhance the recovery of the lumbopelvic muscles.

## Methods

### Study Protocol

After providing written informed consent, 24 individuals participated in the Artificial Gravity Bed Rest—European Space Agency (AGBRESA) study, a joint campaign between ESA, NASA, and DLR, conducted at the “envihab” facility in Cologne, Germany. The experimental procedures were approved by the ethics committee of the Northern Rhine Medical Association (Düsseldorf, Ärztekammer Nordrhein, No. 2018143) and registered at the German Clinical Trials Register (No. DRKS00015677).

This single-center randomized controlled study was initially intended to have an equal number of male and female participants. However, due to some female participants withdrawing from the study, the final cohort consisted of 8 females and 16 males. The study followed the “International Guidelines for Standardization of Bed Rest Studies in the Spaceflight Context” (Sundblad et al., 2016), in which all detailed aspects of bed rest studies are described, including inclusion and exclusion criteria, participant position, monitoring of activities, and medical care.

**FIGURE 1:**
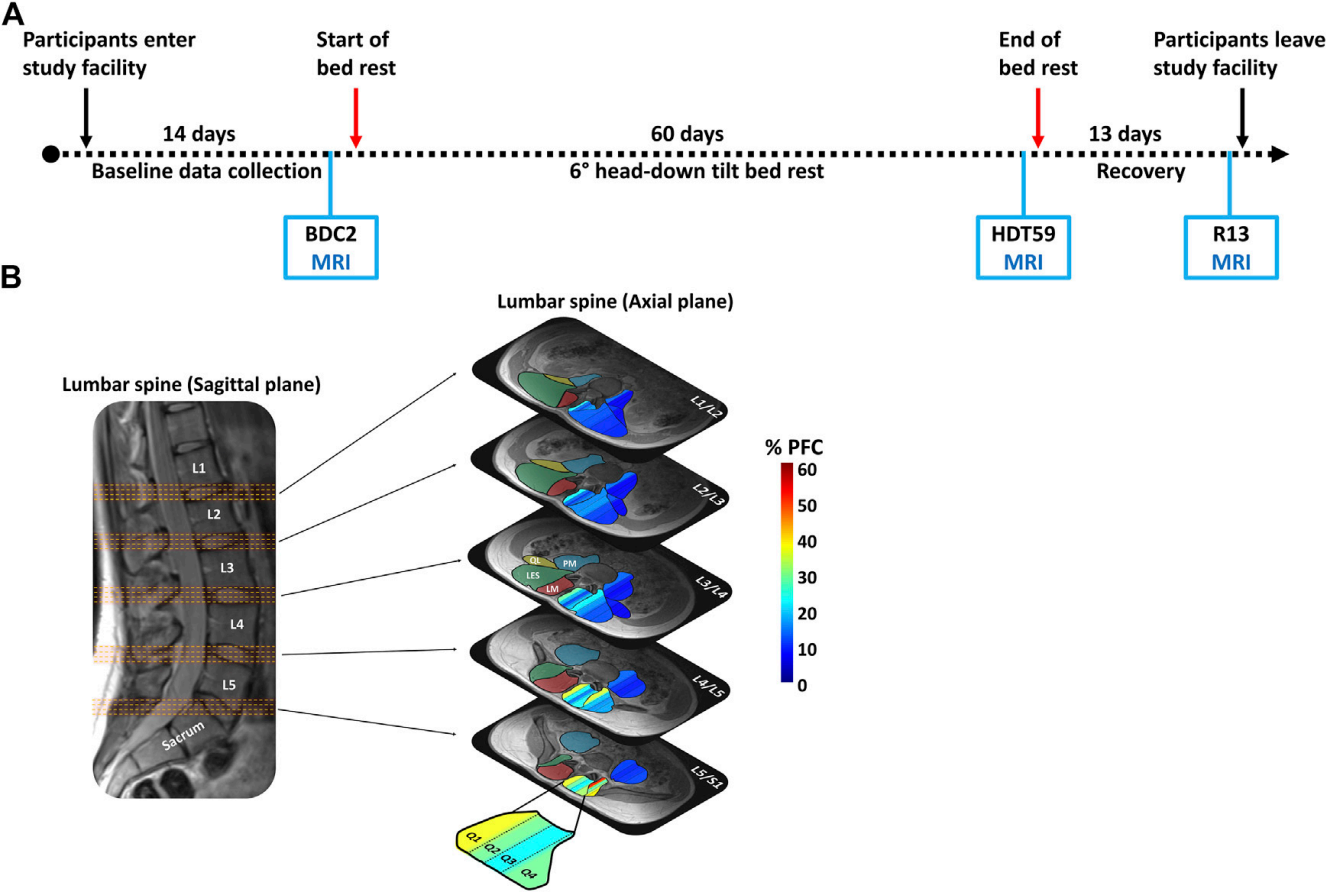
**(A)** Time schedule of each of the two campaigns. Magnetic resonance imaging was collected 2 days before the beginning of the bed rest period (BDC2), day 59 of the head-down tilt bed rest (HDT59), and day 13 of the recovery period (R13). **(B)** Five groups of four axial slices were identified from the sagittal image for each of five lumbar intervertebral disc levels (L1/L2, L2/L3, L3/L4, L4/L5, L5/S1) to obtain the images used for measurement. The muscle volume of the lumbar multifidus (LM—red shaded area), lumbar erector spinae (LES—green shaded area), quadratus lumborum (QL—yellow shaded area), and psoas major (PM—blue shaded area) was calculated. Paraspinal muscle fat content (PFC) was automatically quartiled from medial to lateral based on equal pixel numbers (Q1, Q2, Q3, and Q4). The color scale represents the percentage of fat content (0%–60%).

The study was divided into two campaigns with 12 participants in each. Each campaign consisted of 14 days of baseline data collection (BDC), 60 days of HDT bed rest, and 13 days of recovery (Figure 1A). The primary goal of the study was to investigate the efficacy of a short-radius centrifuge to create artificial gravity (AG), as a countermeasure for physiological adaptations provoked by HDT bed rest. The results of the AG analysis did not show any evident protective effects of the AG intervention on lumbopelvic (De Martino et al., 2021b; 2021c). The secondary goal was to investigate the efficacy of the SR program and SR + FRED programs for the reconditioning of lumbopelvic muscles following HDT bed rest.

As part of the primary goal of the study, participants were randomized to three intervention groups: a group that underwent 30-min of continuous centrifugation/day (cAG), a group that underwent six sets of 5-min centrifugation/day (iAG) interspersed by 3-min rest, and a group that was not exposed to AG (CTRL) (Frett et al., 2020). At the same time, participants were randomly assigned 1:1 to SR or SR + FRED program. This design ensured equal representation of the three groups during bed rest (cAG, iAG, and CTRL) in each reconditioning group (SR and SR + FRED). Sex, age, height, and weight of the two reconditioning groups were comparable and did not show any statistically significant difference (SR group—4 females, 8 males, 35 ± 10 years, 174 ± 9 cm, 74 ± 11 kg; SR + FRED group—4 females, 8 males, 32 ± 9 years, 176 ± 7 cm, 75 ± 9 kg).

### Reconditioning Protocols

Daily ambulatory activity (around the ward) was supplemented during the recovery phase by two reconditioning programs: SR or SR + FRED program (Table 1). The SR program consisted of seven sessions of dynamic stability, coordination, postural stability, and stretching exercises performed for 1 h from R + 4 to R + 11, with a rest day on R + 8. Bodyweight resisted exercises were incorporated in the regime, but additional resistance was not added. The SR program included exercises such as single-leg standing with eyes open and closed, double and single-leg squats, standing on a foam pad and stepping over/walking around obstacles. The program was complemented by passive stretching of the hamstring, gluteal, lumbar and quadriceps muscle groups, as well as individual exercises tailored to the needs of the participant if specific deficits were observed. Exercises were progressed by increasing the number of repetitions, and the exercise sessions were supervised by an expericed specialist and a physiotherapist.

**TABLE 1:** Reconditioning programs for SR group (*N* = 12) and SR + FRED group (*N* = 12).

	R1	R2	R3	R4	R5	R6	R7	R8	R9	R10	R11	R12	R13
SR group				SR	SR	SR	SR		SR	SR	SR		
SR + FRED group	FRED	FRED	FRED	SR + FRED	SR + FRED	SR + FRED	SR + FRED	FRED	SR + FRED	SR + FRED	SR + FRED	FRED	FRED

In addition to the SR, 12 participants performed 13 training sessions of FRED from R1 to R13. Participants were instructed to stand with their knees slightly bent with a relaxed upper body posture while keeping the lower limbs moving in a slow, controlled manner with minimal variability in their movement speed throughout the cycle (Weber et al., 2018). Visual feedback of the rotational frequency of the footplates was provided on a screen in front of the participant, who was asked to maintain a frequency of 0.42 revolutions per second with as constant a rotational frequency as possible throughout each complete rotation (Weber et al., 2018). The FRED training time was increased incrementally for the first 5 days from 6 to up to 25 min of training, in 3–5 min intervals. Between R7 and R13, participants completed 25 min of FRED exercise each day (Lindsay et al., 2020).

### Assessment of Muscle Volume and Paraspinal Muscle Fat Content

Magnetic Resonance Imaging was performed 2 days before the bed rest period (BDC2), on the 59th day of HDT bed rest (HDT59), and on the 13th day of rehabilitation (R13) (Figure 1A). A 3-Tesla Magnetom Vision system (Siemens, Erlangen, Germany) was used to collect the MRIs. Participants were positioned in supine lying on the scanning table with their knees and hips supported in slight flexion by a pillow. A set of 64 transverse images was acquired from the level of the T12 vertebra to the sacrum (T1-weighted Dixon sequence, total acquisition time = 5 min; slice thickness = 4 mm; distance factor = 20%, TR = 7.02 ms, TE1 = 2.46 ms, TE2 = 3.69 ms, flip angle = 5 deg; field of view = 400 mm × 400 mm at 1.0 mm × 1.0 mm pixel size). Fat and water in-phase and out-of-phase images were collected, and fat (F) and water (W) suppression images were reconstructed. The lumbopelvic muscles’ regions of interest (ROI) were manually traced using a semi-automated Matlab-based program (Mhuiris et al., 2016; Crawford et al., 2019). The custom-built Matlab (Natick, MA, United States) program automatically divided the ROI into quarters of equal areas from medial (Q1) to lateral (Q4) based on the pixel number (Abbott et al., 2018; Crawford et al., 2019). The PFC was calculated as the ratio of pixel intensities from the F and W images:

$$\text{PFC}=\frac{\text{F}}{\left(\text{W }+\text{ F}\right)}*100$$

Five groups of four axial slices were identified for each of five lumbar IVDs (L1/L2, L2/L3, L3/L4, L4/L5, L5/S1) to obtain the axial images used for measurement of muscle volume and PFC (Figure 1B). Bilateral muscle volume and PFC measurements (whole muscle and quartiles) of the LM, LES, QL, and PM muscles were extracted from each axial slice (Abbott et al., 2018; Crawford et al., 2019). Measurements were averaged for the four slices at each lumbar region and the left and right sides. The reliability of quantification of muscle volume and PFC of the lumbopelvic muscles in the axial plane has been previously demonstrated (De Martino et al., 2021b).

### Statistical Analyses

SPSS software (Version 25, IBM, Chicago, IL, United States ) was used for statistical analysis. All results are reported as means (standard deviation, SD), and statistical significance was set at the (2-sided) 0.05 level. Visual examination (histograms and Q-Q plots) and Shapiro–Wilk tests were used to assess the normality of outcomes. Muscle volumes and PFC of the whole muscle at the level of each IVD (L1/L2, L2/L3, L3/L4, L4/L5, and L5/S1) for the LM, LES, PM, and QL muscles were analyzed using a two-way mixed-model ANOVA with Group (SR and SR + FRED–between-group factor) and Time (BDC, HDT59, and R13—within-subject factor). Furthermore, PFC at each IVD level for the LM, LES, PM, and QL muscles were analyzed using a three-way mixed-model ANOVA with Group (SR and SR + FRED—between-group factor), Time (BDC, HDT59, and R13—within-subject factor), and Quartile (Q1, Q2, Q3, and Q4—within-subject factor). Interactions between all factors were included in both models. The Greenhouse–Geisser approach was used to correct for violations of sphericity, and effect sizes (partial eta-squared: η_2_^partial^) were calculated. Pairwise comparisons were performed using Bonferroni method to correct for multiple comparisons, and corresponding 95% confidence intervals were generated (Bonferroni adjusted).

## Results

### Lumbopelvic Muscle Volumes

**FIGURE 2:**
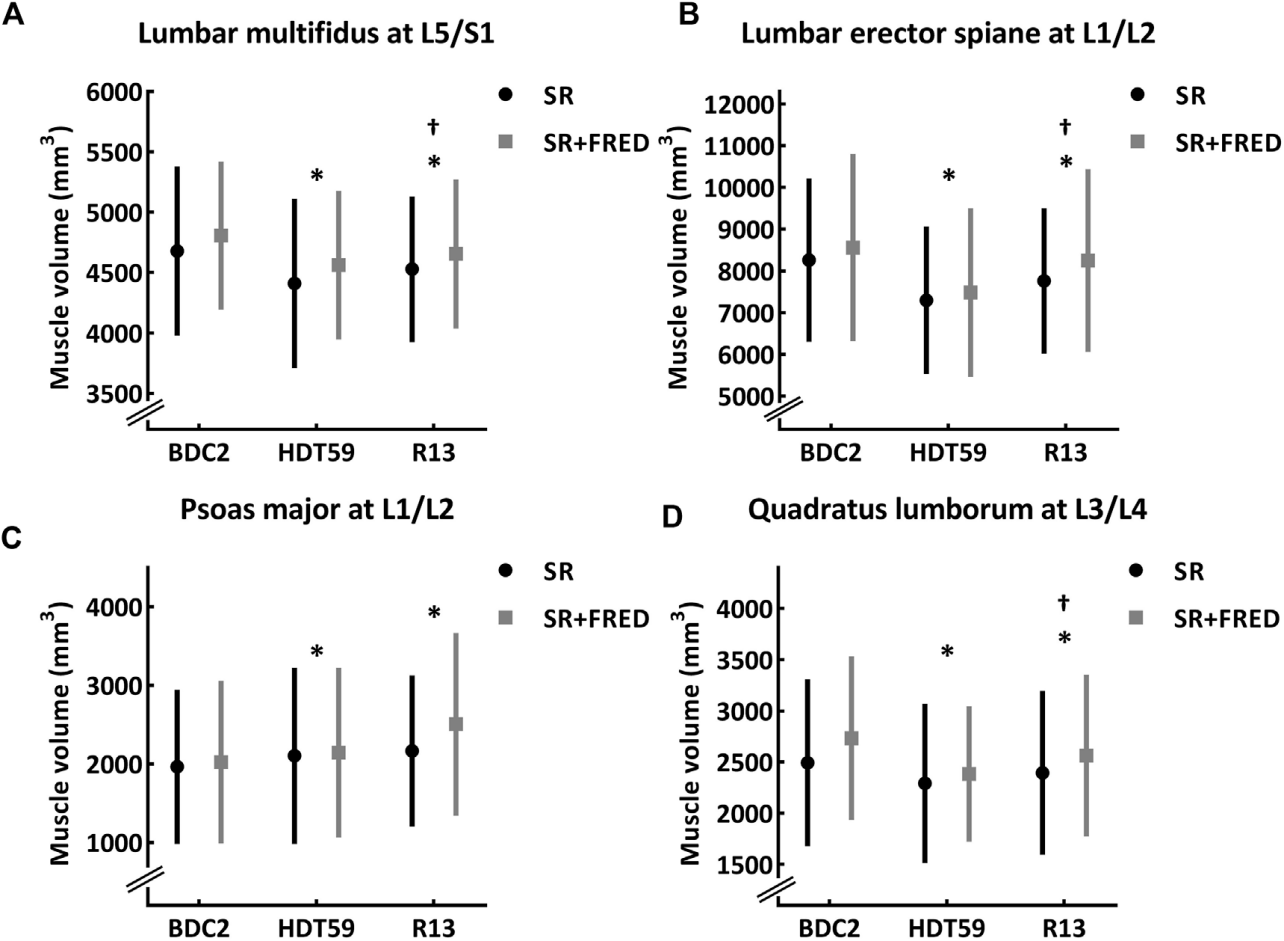
Morphology of the lumbar multifidus, lumbar erector spinae, psoas major, and quadratus lumborum muscles at 2 days before the beginning of the bed rest period (BDC2), day 59 of head-down tilt bed rest (HDT59), and day 13 of recovery period (R13) for participants in the Standard Reconditioning (SR, *n* = 12) and Standard Reconditioning supplemented with Functional Re-adaptive Exercise Device (SR + FRED, *n* = 12). The black line is SR group and the light gray line is SR + FRED group. The group means are a filled circle (SR group) or a filled square (SR + FRED group), and the vertical line is the standard deviation. **(A)** Muscle volume of the lumbar multifidus at L5/S1. **(B)** Muscle volume of the lumbar erector spinae at L1/L2. **(C)** Muscle volume of the psoas major at L1/L2. **(D)** Muscle volume of the quadratus lumborum. The group means are a filled circle (SR group), and the vertical line is the standard deviation. *Significantly different from BDC2 within the group (*p* < 0.05). ^†^Significantly different from HDT59 within the group (*p* < 0.05). SR, Standard Reconditioning; SR + FRED, Standard Reconditioning supplemented with Functional Re-adaptive Exercise Device; BDC, Baseline data collection; HDT, Head-down tilt; R, Recovery.

The results of the two-way mixed-model ANOVA revealed a main effect of Time for the LM muscle volume at the L2/L3, L3/L4, L4/L5, and L5/S1 IVD levels (all—F ≥ 5, *p* < 0.05). Pairwise comparisons showed that the average reduction in LM muscle volume was 6.8% ± 3.7% (−251.6 ± 153.1 mm^3^) at the end of bed rest compared with baseline values (all—*p* < 0.05). In the recovery phase, the LM muscle volume returned towards baseline values at the levels of the L2/L3 (*p* = 0.706; −42.1 mm^3^ with a 95% CI of [−160.3, 57.7]) and L3/L4 IVDs (*p* = 1.000; −26.8 mm^3^ with a 95% CI of [−155.2, 101.7]). By contrast, the LM muscle volume remained smaller than baseline values at the levels of L4/L5 (*p* = 0.014; −203.0 mm^3^ with a 95% CI of [−369.6, −36.4]) and L5/S1 IVDs (*p* = 0.004; −150.7 mm^3^ with a 95% CI of [−258.2, −43.3]) (Table 2 and Figure 2A).

**TABLE 2:** Mean (± standard deviation) muscle volume in mm^3^ from SR (*N* = 12) and SR + FRED (*N* = 12) at BDC2, HDT59, and R13.

Intervertebral Disc Level							
Muscle	Group	Time	L1/L2	L2/L3	L3/L4	L4/L5	L5/S1
Lumbar multifidus	SR	BDC	1310 ± 397	2013 ± 513	3274 ± 819	4771 ± 838	4805 ± 613
		HDT59	1268 ± 387	1911 ± 474^a^	3039 ± 754^a^	4404 ± 783^a^	4561 ± 615^a^
		R13	1242 ± 428	1971 ± 527	3384 ± 954^b^	4566 ± 854^a^ ^,^ ^b^	4655 ± 617^a^ ^,^ ^b^
	SR + FRED	BDC	1256 ± 355	1991 ± 487	3058 ± 698	4559 ± 862	4679 ± 700
		HDT59	1192 ± 329	1874 ± 492^a^	2820 ± 689^a^	4104 ± 864^b^	4410 ± 702^a^
		R13	1282 ± 327	1930 ± 545	3001 ± 954^b^	4358 ± 894^a^ ^,^ ^b^	4528 ± 602^a^ ^,^ ^b^
Lumbar erector spinae	SR	BDC	8261 ± 1956	8337 ± 1839	7265 ± 1433	5324 ± 725	2177 ± 606
		HDT59	7298 ± 1768^a^	7263 ± 1434^a^	6470 ± 1220^a^	5299 ± 743	2390 ± 721^a^
		R13	7759 ± 1741* ^,^ ^†^	7827 ± 1504^a^ ^,^ ^b^	6895 ± 1258^†^	5118 ± 672	2124 ± 617
	SR + FRED	BDC	8559 ± 2246	8695 ± 2046	7601 ± 1712	5248 ± 928	2384 ± 674
		HDT59	7483 ± 2022^a^	7832 ± 1917^a^	6980 ± 1689^a^	5202 ± 864	2621 ± 728^a^
		R13	8249 ± 2191^a^ ^,^ ^b^	8571 ± 2132^a^ ^,^ ^b^	7595 ± 1814^b^	5223 ± 943	2414 ± 765
Psoas major	SR	BDC	1963 ± 979	3619 ± 1405	4939 ± 1539	5879 ± 1616	5679 ± 1586
		HDT59	2104 ± 1120^a^	3697 ± 1389	4931 ± 1562	5828 ± 1726	5709 ± 1658
		R13	2165 ± 959^a^	3777 ± 1221	5014 ± 1379	5916 ± 1483	5665 ± 1420
	SR + FRED	BDC	2021 ± 1035	4358 ± 1491	5897 ± 1779	6799 ± 1797	6456 ± 1666
		HDT59	2143 ± 1078^a^	4468 ± 1694	5971 ± 1793	6829 ± 1805	6418 ± 1663
		R13	2505 ± 1163^a^	4589 ± 1466	6089 ± 1790	6993 ± 1820	6510 ± 1625
Quadratus lumborum	SR	BDC	1062 ± 401	1798 ± 755	2493 ± 813	—	—
		HDT59	1003 ± 380	1658 ± 702^a^	2291 ± 777^a^	—	—
		R13	990 ± 445	1720 ± 732	2392 ± 800^a^ ^,^ ^b^	—	—
	SR + FRED	BDC	1125 ± 477	1919 ± 710	2733 ± 799	—	—
		HDT59	1015 ± 420	1660 ± 600^a^	2383 ± 659^a^	—	—
		R13	1111 ± 471	1716 ± 781	2561 ± 789^a^ ^,^ ^b^	—	—

a Pairwise comparisons (Bonferroni adjusted) relative to baseline (BDC) value (*p* < 0.05).

b Pairwise comparisons (Bonferroni adjusted) relative to the end of head-down tilt (HDT59) bed rest value (*p* < 0.05).

SR, Standard Reconditioning; SR + FRED, Standard Reconditioning supplemented with Functional Re-adaptive Exercise Device; BDC, Baseline data collection; HDT, Head-down tilt; R, Recovery.

A main effect of Time was found for the volume of the LES muscles at the L1/L2, L2/L3, L3/L4, and L5/S1 IVD levels (all—F > 5, *p* < 0.05). At the end of bed rest, the average reduction in LES muscle volume was 11.2% ± 5.5% (−926.6 ± 511.2 mm^3^) compared with baseline values (all – *p* < 0.05), except for L5/S1 IVD level where it increased (*p* = 0.002). In the recovery phase, the LES muscle volume returned towards baseline values at the L3/L4 (*p* = 0.207; −187.8 mm^3^ with a 95% CI of [−442.2, 66.7]) and L5/S1 (*p* = 1.000; 11.5 mm^3^ with a 95% CI of [−187.8, 210.9]) IVD levels, but remained smaller than baseline values at the level of the L1/L2 (*p* = 0.002, −405.3 mm^3^ with a 95% CI of [−667.0, −143.6]) and L2/L3 (*p* = 0.021, −316.9 mm^3^ with a 95% CI of [−592.2, −41.6]) IVDs (Table 2 and Figure 2B).

A Time effect was detected for the PM muscle volume at the L1/L2 IVD level (F = 5.5, *p* = 0.024). At the end of bed rest, the PM muscle volume increased compared with the baseline value (*p* = 0.010, 211.3 mm^3^ with a 95% CI of [45.8, 376.7]) and was still greater (*p* = 0.001, 293.0 mm^3^ with a 95% CI of [132.2, 453.2]) in the recovery phase (Table 2 and Figure 2C).

A Time effect was revealed for the QL muscle volume at the L2/L3 and L3/L4 IVD levels (both—F > 5, *p* < 0.05). The average reduction in QL muscle volume was 10.5 ± 8.3% (−237.7.6 ± 208.1 mm^3^) compared with baseline values (both–*p* < 0.05). In the recovery phase, the QL muscle volume showed a strong tendency to return towards baseline values at the L2/L3 IVD level (*p* = 0.050, −140.5 mm^3^ with a 95% CI of [−281.0, −0.1]) but remained smaller than baseline values at L3/L4 (*p* = 0.042, −135.7 mm^3^ with a 95% CI of [−267.6, −3.8]) IVD level (Table 2 and Figure 2D).

There was no difference between Groups or interactions between Time*Group at any IVD level for these muscles.

All detailed statistical results of the two-way mixed-model ANOVA are reported in Supplementary Table S1.

### Paraspinal Muscle Fat Content

The results of the two-way mixed-model ANOVA revealed a main effect of Time for the LM muscle PFC at the L2/L3, L3/L4, L4/L5, and L5/S1 IVD levels (all—F > 10, *p* < 0.001). Pairwise comparisons showed that the average increase in LM muscle PFC (average L2/L3, L3/L4, L4/L5, and L5/S1 IVD levels) was 3.5% ± 1.3% at the end of bed rest compared with baseline values (all—*p* < 0.01). In the recovery phase, the LM muscle PFC returned towards baseline values at the levels of the L2/L3 (*p* = 1.000; 0.1% with a 95% CI of [−0.9, 1.2]), L3/L4 IVDs (*p* = 0.106; 0.7% with a 95% CI of [−0.1, 1.5]), and L5/S1 IVDs (*p* = 0.306; 0.7% with a 95% CI of [−0.3, 1.7]). By contrast, the LM muscle PFC remained higher than baseline values at the levels of L4/L5 (*p* = 0.046; 1.0% with a 95% CI of [0.1, 2.1]).

A main effect of Time was also found for the PFC of the LES muscles at the L1/L2, L2/L3, L3/L4, L4/L5, and L5/S1 IVD levels (all—F > 5, *p* < 0.05). Pairwise comparisons showed that the average increase in LES muscle PFC (average all IVD levels) was 2.1 ± 0.4% at the end of bed rest compared with baseline values (all—*p* < 0.01). In the recovery phase, the LES muscle PFC returned towards baseline values at the levels of the L1/L2 (*p* = 1.000; 0.1% with a 95% CI of [−0.6, 0.7]), L2/L3 (*p* = 0.215; 0.4% with a 95% CI of [−0.1, 0.9]), L3/L4 (*p* = 0.067; 0.8% with a 95% CI of [−0.1, 1.5]), L4/L5 IVDs (*p* = 0.430; 0.9% with a 95% CI of [−0.7, 2.6]), and L5/S1 IVDs (*p* = 1.000; 0.8% with a 95% CI of [−1.4, 2.9]).

No significant changes in Time or Group or Time*Group interactions were found in QL and PM muscle PFC (all—F < 5, *p* > 0.05). All detailed statistical results of the two-way mixed-model ANOVA are reported in Supplementary Tables S2, S3.

**FIGURE 3:**
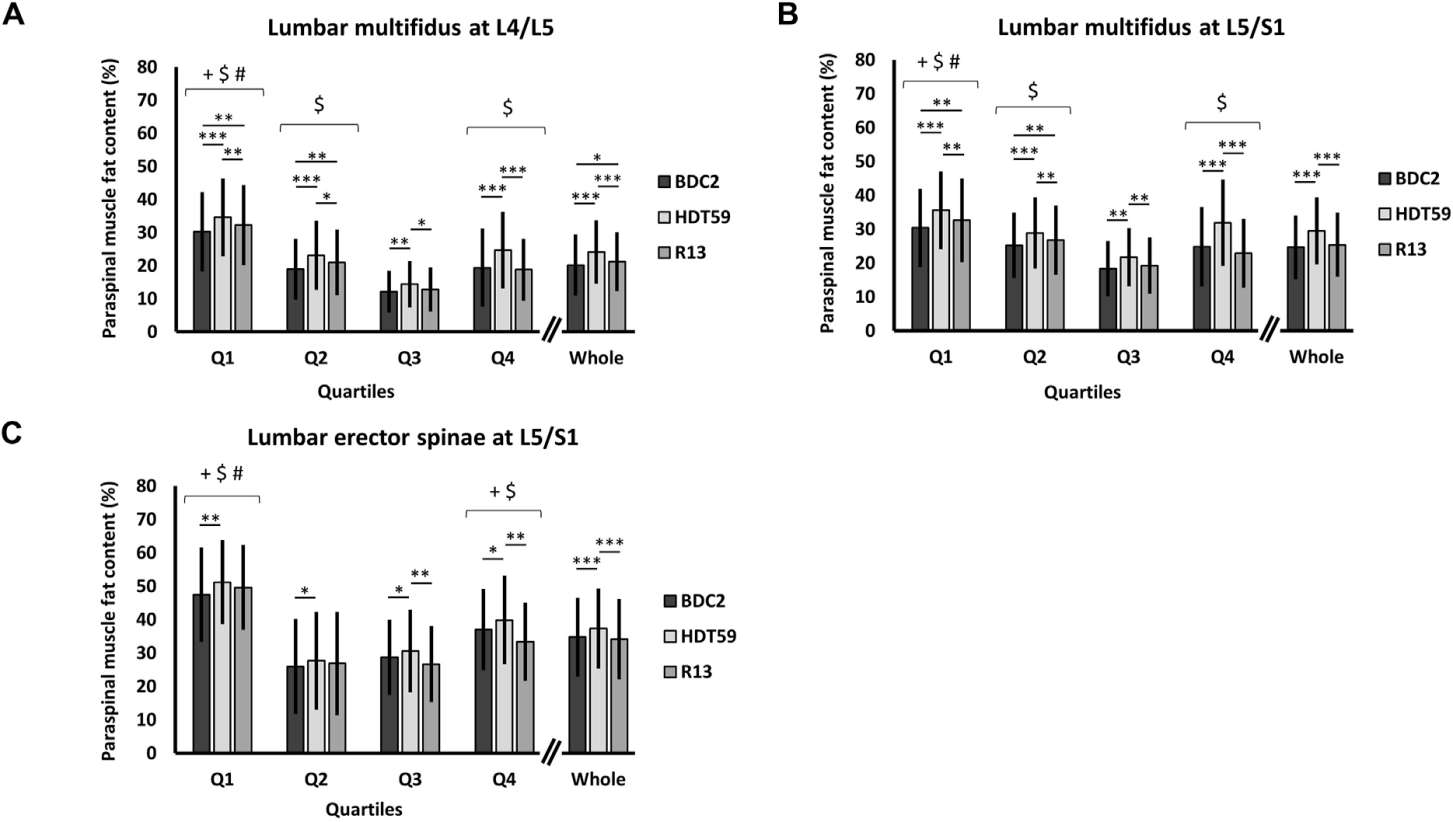
Group data (mean ± standard deviation, *n* = 24) of paraspinal muscle fat content (%) in Q1, Q2, Q3, Q4, and whole muscle 2 days before the beginning of the bed rest period (BDC2), day 59 of head-down tilt bed rest (HDT59), and day 13 of the recovery period (R13). The black bar is BDC2, the light gray bar HDT59, and the dark gray bar R13. **(A)** Paraspinal muscle fat content in the lumbar multifidus muscle at L4/L5. **(B)** Paraspinal muscle fat content in the lumbar multifidus muscle at L5/S1. **(C)** Paraspinal muscle fat content in the lumbar erector spinae at L5/S1. Main effect of Quartile: Pairwise comparisons (Bonferroni adjusted) ^+^ *p* < 0.05 compared with Q2; ^$^ *p* < 0.05 compared with Q3; ^#^ *p* < 0.05 compared with Q4. Time*Quartile interaction: Pairwise comparisons (Bonferroni adjusted) *p < 0.05; **p < 0.01; ***p < 0.001. BDC, Baseline data collection; HDT, Head-down tilt; R, Recovery.

The results of the three-way mixed-model ANOVA revealed a Time*Quartile interaction for the LM muscle PFC at the L4/L5 and S1/L5 IVD levels (both—F > 5, *p* < 0.01). At the end of the bed rest, pairwise comparisons showed that the average increase in PFC in all quartiles (average L4/L5 and S1/L5 IVD levels) was 4.2 ± 2.1% compared with baseline values (all—*p* < 0.01). In the recovery phase, PFC returned towards baseline values in Q3 (L4/L5—*p* = 0.189, 0.7% with a 95% CI of [−0.2, 1.7]; L5/S1—*p* = 0.407, 0.9% with a 95% CI of [−0.6, 2.4]) and Q4 (L4/L5—*p* = 1.000, −0.6% with a 95% CI of [−3.3, 2.1]; L5/S1—*p* = 0.247, −1.9% with a 95% CI of [−4.5, 0.8]), but remained higher than baseline values in Q1 (L4/L5—*p* = 0.001, 2.0% with a 95% CI of [0.7, 3.2]; L5/S1–*p* = 0.001, 2.2% with a 95% CI of [1.1, 3.3]) and Q2 (L4/L5–*p* = 0.001, 2.1% with a 95% CI of [0.8, 3.4]; L5/S1–*p* = 0.002, 1.5% with a 95% CI of [0.5, 2.5]) (Figures 3A,B).

For the LES muscle, the three-way mixed-model ANOVA revealed a Time*Quartile interaction at the L5/S1 IVD level (F = 6.3, *p* = 0.001). At the end of bed rest, the average increase in PFC in all quartiles was 2.6 ± 2.3% compared with baseline values (all—*p* < 0.05). In the recovery phase, PFC was not different to the baseline values in all quartiles (all—*p* > 0.05), but a significant statistical reduction was only found in Q3 (*p* = 0.001, −3.8% with a 95% CI of [−6.2, −1.6]) and Q4 (*p* < 0.001, −6.5% with a 95% CI of [−9.8, −3.1]) compared with the end of the bed rest (Figure 3C).

The three-way mixed-model ANOVA revealed the main effects of Time for the LM and LES muscles at all IVD levels (F ≥ 5, *p* < 0.05). PFC in the LM and LES muscles increased from the baseline values to the end of the bed rest (all—*p* < 0.05). In the recovery phase, most of these variables returned towards baseline values (all—*p* < 0.05), except for the LM muscle at the L4/L5 and L5/S1 IVD levels, where PFC remained significantly greater (both—*p* < 0.05).

Main effects of Quartile were detected for all lumbopelvic muscles at all IVD levels (F > 10, *p* < 0.001). The highest PFC values were constantly detected in the medial regions (Q1) and progressively decreased towards the lateral regions (Q4). However, the lowest PFC values were frequently detected in Q2 or Q3.

A main effect of Group was found for the LM and LES muscles at almost all IVD levels (F ≥ 5, *p* < 0.05; Supplementary Tables S2, S3), with higher values of PFC in SR than SR + FRED group.

There were no interactions found for Group*Time, Quartile*Group or Time*Quartile*Group at any lumbar level.

All detailed statistical results of three-way mixed-model ANOVA and PFC values are reported in Supplementary Tables S4–S7.

## Discussion

This study examined the recovery of muscle atrophy and the accumulation of PFC in the lumbopelvic muscles following reconditioning programs of 2 weeks duration adopted after 60-day of HDT bed rest. The present results showed that reconditioning programs partially reversed the changes in the volumes of the LM, LES, QL, and PM muscles, but not the LM at L4/5 and L5/S1. In addition to the lack of change in the volume of the LM muscle at the lower vertebral levels, localized accumulation of lipids in the medial regions of this muscle was still evident at the end of the reconditioning period compared with baseline values. The application of FRED, in addition to the SR program, did not lead to additional benefits.

### Muscle Volumes After Reconditioning

In line with prior bed rest and spaceflight studies (Belavý et al., 2010; Bailey et al., 2018), the current analysis confirmed that muscle atrophy of the lumbopelvic muscles was induced by prolonged bed rest, with the greatest atrophy observed in the LM muscle at the levels of L4/L5 (8.9 ± 3.0%), in the LES muscle at the levels of L1/L2 (13.6 ± 2.1%), and QL muscles at the levels of L3/L4 (10.6 ± 6.6%). These lumbar levels, in general, represent the anatomical locations where the muscles have the greatest cross-sectional areas, which may explain why the potential for atrophy is greatest at these levels. 2 weeks of reconditioning were insufficient to fully restore the volumes of the muscles assessed to their baseline values. After reconditioning, muscle volumes of the LM at L4/L5 level, LES at L1/L2 level, and QL at L3/L4 level were still decreased by 4.4 ± 6.2%, 4.5 ± 5.3%, 5.3 ± 10.6%, respectively, when compared with baseline. This decrease was greater than the 1.6%, 2.8%, and 1.2% reductions in LM muscle between L4 and L5 levels (averaged), LES muscle between L1 and L2 levels (averaged), and QL muscle between L3 and L4 levels (averaged), respectively, reported after reconditioning in a previous 60-day HDT bed rest, where motor control training, trunk flexor and general strength programs were applied (Hides et al., 2011).

In contrast, the PM muscle increased by 17.1% ± 13.4% at L1/L2 level after reconditioning in the current study, which is greater than the maximal 6.3% increase at L4/L5 level reported in the previous bed rest study (Hides et al., 2011). This discrepancy in results may be explained by the different types of reconditioning programs used in these HDT bed rest studies. While the current study involved the performance of functional bodyweight exercises without resistance, the previous study also progressed to resisted weight-bearing exercises using the TheraBand with the lumbar spine held in a neutral position (Hides et al., 2011). Some methodological differences, such as the use of alternate imaging equipment and slice thickness, could also potentially explain the disparities between studies. The two studies also differed in terms of the sex of the participants. In contrast to the prior study (Hides et al., 2011), which exclusively included males, the current study also included eight female participants.

With respect to reconditioning of astronauts after exposure to microgravity, a previous study showed that the size of the LM muscle decreased by 9.7% between the L4 and L5 vertebral levels (averaged) after 6 months on the International Space station. This atrophy was reversed after 2 weeks of intense and individualized reconditioning (Hides et al., 2020). The ESA astronaut reconditioning program begins with voluntary, isolated contractions to re-educate lumbar muscle recruitment and progresses quickly to restore the lumbar lordosis and normal movement patterns in upright standing (Lambrecht et al., 2017). The progression to resistance and endurance exercises can occur quite rapidly, as soon as postural lumbar alignment and optimal movement patterns are regained (Lambrecht et al., 2017). Resisted strength exercises (e.g., squats with loads) start from recovery day 5, and endurance exercises (e.g., plank exercises) begin from recovery day 11 (Lambrecht et al., 2017). Astronauts also undergo longer daily reconditioning sessions (90 min per day) (Lambrecht et al., 2017). Several differences between astronauts and bed rest participants also require consideration. Astronauts are well prepared before spaceflight, can freely move around in microgravity, and perform approximately 2-h of daily exercise, including loaded exercises on the Advanced Resistive Exercise Device whilst in the microgravity environment (Petersen et al., 2016). Together, these factors may contribute to a faster recovery of the lumbopelvic muscles in astronauts after landing than observed following prolonged HDT bed rest, despite the longer duration of space missions. However, caution is needed when comparing results from HDT bed rest and spaceflight since durations between actual space flight and space flight analogs such as bed rest may vary significantly (i.e., for the present study, exposure to 60 days bed rest vs. 6–7 months microgravity exposure during long duration ISS missions).

### Paraspinal Muscle Fat Content After Reconditioning

The current study has provided novel insights into the effects of reconditioning of PFC in the LM muscles. No previous studies have investigated the effects of reconditioning on PFC following spaceflight or prolonged HDT bed rest. This aspect may be an important consideration given that the accumulation of PFC in the LM muscle appears to impact the capacity to meet functional demands to control the spine (Hodges et al., 2015; Teichtahl et al., 2015).

In the current investigation, not all regions of the LM muscle recovered to their baseline values. While the lateral regions of LM muscle returned to their baseline values, the medial regions at the levels of the L4/L5 and L5/S1 IVDs showed more than 2% of PFC accumulation. It is also important to note that PFC was higher adjacent to the medial and lateral aspect of muscle, as shown by our measurements at BDC2, suggesting that fascia, perimuscular connective tissues, epi-peri and endomysial fat deposits may contribute to these higher values. A study on changes in paraspinal muscle fat following free weight-based resistance training for people with chronic LBP also showed that exercise reduced PFC of the LM and LES muscles at the L3/4 and L4/5 vertebral levels but not at L5/S1 (Welch et al., 2015). The L5/S1 vertebral level had higher percentages of PFC pre-intervention, and the investigators proposed that muscles with a higher percentage of PFC may be more resilient to change in response to exercise or alternatively that the loading may have been distributed unevenly with decreased loading on the LM muscle in that region.

The findings of the current investigation also indicated an inhomogeneous recovery of PFC, which could possibly be explained by the heterogeneous architectural structure and function of the LM muscle (Macintosh and Bogduk, 1986; Macintosh et al., 1986). Dissection studies have revealed that the LM muscle is composed of both long and short fibers. The long fibers arise from the spinous process of each lumbar vertebra and cross up to five vertebral segments to insert on the ilia and sacrum (Macintosh and Bogduk, 1986; Macintosh et al., 1986). These long fascicles have a great moment arm and are suited to resisting flexion of the lumbar spine in upright standing (Macintosh and Bogduk, 1986; Moseley et al., 2003) and controlling the lumbar lordosis as their line of action falls behind the lumbar curve (Moseley et al., 2003). In the current study, the lateral region of the LM muscle at the L4/L5 and L5/S1 levels would represent the distal portion of the long fibers originating from upper lumbar levels. Since this muscle region returned to baseline values, one could hypothesize that the reconditioning programs used in the current study sufficiently stimulated these fibers. By contrast, the short fibers of LM originate from the laminae of the lumbar vertebrae and insert into the mamillary processes of two vertebral segments below (Macintosh et al., 1986). These fibers have a small moment arm, and are suited to exert a focal increase in spinal stiffness in functional loading tasks (Moseley et al., 2003). In the current study, the medial region at the L4/L5 and L5/S1 intervertebral levels would represent the short fibers at lower vertebral levels. It is possible that these short fibers were not sufficiently engaged in the exercises selected during the reconditioning period.

Increased muscle loading and mechanical stretch of muscle fibers have demonstrated downregulation of adipogenic transcription factors (Akimoto et al., 2001; Kook et al., 2008) and increased expression of factors that inhibit myoblast transdifferentiation to adipocytes (Akimoto et al., 2005). In people with chronic low back pain, resisted weight-bearing exercises in a neutral position of the spine have been shown to induce hypertrophy and reduce PFC of the lumbar paraspinal muscles (Welch et al., 2015). Consequently, progressive resisted weight-bearing exercises performed while maintaining a lumbar lordosis may play a crucial role in recovering muscle properties after lumbar spine deconditioning.

### Standard Reconditioning Program Supplemented With FRED

Contrary to our hypothesis, the application of FRED, in addition to the SR program, did not produce any evident additional benefits for the outcomes measured in the present study that are linked to lumbopelvic muscle recovery. The SR program was characterized by bodyweight exercises focused on static and dynamic balance, coordination, and postural control. As the SR program targeted weight-bearing muscles, the improvements that were observed (increasing muscle volumes and decreasing PFC post bed rest) were most likely due to the recruitment of the muscles in the bodyweight exercises selected.

Exercise on the FRED involves a combination of weight-bearing while holding a neutral, upright sagittal spinal alignment over an unstable base of support at the feet. Previous studies have demonstrated that exercise on the FRED recruited the LM muscle (Debuse et al., 2013; Winnard et al., 2017a). Compared to both overground (Caplan et al., 2014) and treadmill (Weber et al., 2017) walking, a more constant low-level activity of the LM muscle has been reported, as well as a more anteriorly tilted pelvic position during exercise on FRED (Winnard et al., 2017b). However, the current results failed to show that supplementation of FRED enhanced the effect of the SR program alone on muscle volume and PFC accumulation, most likely because of the absence of progressive external loads. This could indicate that implementation of the FRED might be useful in the early stages of reconditioning, requiring initial recruitment of lumbopelvic muscles after bed rest, but progression to resistance training may be required in later stages of the rehabilitation programs after bed rest or spaceflight.

### Relevance for Patients and Astronauts

The current results support previous studies showing that a prolonged period of axial unloading induces lumbar spine deconditioning in healthy individuals (Burkhart et al., 2019; Bailey et al., 2021). Greater amounts of PFC in lumbar musculature have been associated with high intensity of low back pain/disability in a community-based population (Kjaer et al., 2007; Teichtahl et al., 2015), and a 16-week resistance training reduced PFC and improved quality of life (Welch et al., 2015). Appropriate reconditioning programs for the lumbar musculature are likely necessary to remediate deconditioning of the lumbar spine after prolonged body unloading, and understanding which exercises recover muscle structure best will help health professionals tailor improved interventions for astronauts, people who are bedridden, extreme sedentary individuals, and people with LBP.

Space Agencies have reset their foci on Moon and Mars missions and crewed deep space exploration missions, first to the Moon and then later, hopefully also to Mars are already on the horizon and are already in preparation (International Space Exploration Coordination Group, 2018). While NASA’s Apollo missions were designed with a direct journey to the Moon (3 days in microgravity) and a short stay on the lunar surface (max accumulated lunar surface extravehicular activity time was around 22 h), future space missions such as the ARTEMIS program will require prolonged exposure to microgravity and hypogravity (NASA technical report, 2020). For instance, lunar gateway missions will take between 30–40 days, and extravehicular activities on the Lunar surface will be performed after extended periods of exposure to microgravity. Depending on the mission scenario, astronauts will spend a few weeks in microgravity and then be exposed to lunar gravity, where they need to perform tasks on their own and extract themselves from the landing vehicle. More extended spaceflights may also preclude the use of devices such as the Advanced Resistive Exercise Device (ARED) due to the large size of the device and severe volume constraints of deep space exploration vehicles. After long-duration missions, it will be essential to identify the most effective interventions that can reverse the deconditioning effects of microgravity and hypogravity on many systems, including the neurovestibular, cardiovascular, hemato-immunological, and musculoskeletal systems (Demontis et al., 2017; Richter et al., 2017). Our results demonstrated that reconditioning programs of 2 weeks duration based on weight-bearing exercises without additional resistance were not sufficient to fully restore the muscle size and PFC of the lumbar musculature.

### Limitation

There are some notable limitations to the current study, similar to previous bed rest studies (Hides et al., 2007; Belavý et al., 2010). Due to the complexity and high cost, small sample sizes limit the opportunity to detect low-moderate effects between the reconditioning programs (type II error), which may be clinically meaningful. Because the groups have a small sample size, only large effect sizes from interventions can be identified. Related to this, many connected and similar outcomes have been extracted from the MRIs, given the limited opportunity to assess these images. A small sample size also increases the risk of type I error, which may explain the main effect of Group found in the current study. At BDC2, the SR group already showed higher PFC values than the SR + FRED group in the LM and LES muscles.

A significant limitation is the absence of a control group that did not engage in a reconditioning program. However, previous studies have shown long-lasting lumbopelvic muscle atrophy after prolonged bed rest (Hides et al., 2007; Belavý et al., 2008), and reconditioning was ethically necessary for all participants. Furthermore, the current study aimed to investigate the difference between the two reconditioning programs rather than the efficacy of reconditioning versus no reconditioning.

A methodological study limitation in the current project is the MRI sequences. Although typical chemical shift MRI sequences are routinely used and offer the opportunity to investigate the relative ratio of fat to water content within individual voxels on an MRI (Crawford et al., 2017), other fibrous non-muscular elements could not be discriminated in the muscles’ regions of interest (Reeder et al., 2012). In the current study, we decided to apply a proposed method to investigate the muscle proprieties of the paraspinal muscles (Crawford et al., 2017) to allow accurate and reliable comparison of paraspinal muscle quality in future studies. However, the application of different techniques to assess paraspinal muscle composition, such as magnetic resonance spectroscopy, may provide additional information on various tissue metabolites (Fischer et al., 2013), provided a homogenous sample is available.

## Conclusion

This study showed that both 2 weeks of reconditioning programs following 60 days of HDT bed rest were insufficient to restore all volumes of lumbopelvic muscles and reverse the accumulation of PFC in the muscles measured to pre-bed rest values. The application of the FRED to the SR programme did not produce any additional benefits.

## Data Availability

The original contributions presented in the study are included in the article/Supplementary Material, further inquiries can be directed to the corresponding author.
