# Supplementary material for: The Effects of Reconditioning Exercises Following Prolonged Bed Rest on Lumbopelvic Muscle Volume and Accumulation of Paraspinal Muscle Fat
Source: Front Physiol. 2022 Jun 14;13:862793. doi: 10.3389/fphys.2022.862793 (PMC9237402; doi:10.3389/fphys.2022.862793)
Supplement: Supplementary file 1 [file DataSheet1.docx]

Supplementary Material

**Supplementary table 1.** Results of the 2-way mixed model ANOVA of muscle volume using Time (BDC2, HDT59, and R13) as within factors, and Group (SR and SR+FRED) as between-group factor. Boldface indicated P < 0.05.

| Intervertebral  disc level | Muscle volume | 2-way mixed model ANOVA | | |
| --- | --- | --- | --- | --- |
|  |  | Time | Group | Time*Group |
| L1/L2 | LM | F_1.5,32.7_=2.7; P=0.095; η^2^_partial_=0.12 | F_1,22_=0.1; P=0.870; η^2^_partial_<0.10 | F_1.5,32.7_=3.5; P=0.052; η^2^_partial_=0.14 |
|  | LES | **F_2,44_=55.3; P<0.001; η^2^_partial_=0.72** | F_1,22_=0.2; P=0.692; η^2^_partial_<0.10 | F_2,44_=1.3; P=0.297; η^2^_partial_<0.10 |
|  | PM | **F_2,44_=15.4; P<0.001; η^2^_partial_=0.41** | F_1,22_=1.7; P=0.201; η^2^_partial_<0.10 | F_2,44_=3.1; P=0.063; η^2^_partial_=0.13 |
|  | QL | F_1.4,31.5_=3.4; P=0.056; η^2^_partial_=0.14 | F_1,22_=0.1; P=0.710; η^2^_partial_<0.10 | F_1.4,31.5_=1.8; P=0.200; η^2^_partial_<0.10 |
| L2/L3 | LM | **F_1.2,27.1_=5.2; P=0.025; η^2^_partial_=0.19** | F_1,22_=0.1; P=0.870; η^2^_partial_<0.10 | F_1.2,27.1_=0.45; P=0.881; η^2^_partial_<0.10 |
|  | LES | **F_2,44_=47.5; P<0.001; η^2^_partial_=0.68** | F_1,22_=0.6; P=0.459; η^2^_partial_<0.10 | F_2,44_=1.8; P=0.176; η^2^_partial_<0.10 |
|  | PM | F_1.8,40.5_=2.8; P=0.089; η^2^_partial_=0.12 | F_1,22_=1.7; P=0.201; η^2^_partial_<0.10 | F_1.8,40.5_=0.1; P=0.875; η^2^_partial_<0.10 |
|  | QL | **F_2,44_=8.7; P=0.001; η^2^_partial_=0.29** | F_1,22_=0.1; P=0.891; η^2^_partial_<0.10 | F_2,44_=1.0; P=0.367; η^2^_partial_<0.10 |
| L3/L4 | LM | **F_1.5,32.8_=18.9; P<0.001; η^2^_partial_=0.46** | F_1,22_=0.7; P=0.489; η^2^_partial_<0.10 | F_1.5,32.8_=2.1; P=0.154; η^2^_partial_<0.10 |
|  | LES | **F_2,44_=29.2; P<0.001; η^2^_partial_=0.57** | F_1,22_=0.7; P=0.413; η^2^_partial_<0.10 | F_2,44_=1.8; P=0.179; η^2^_partial_<0.10 |
|  | PM | F_1.9,41.2_=3.5; P=0.064; η^2^_partial_=0.15 | F_1,22_=2.2; P=0.153; η^2^_partial_<0.10 | F_1.9,41.2_=0.4; P=0.690; η^2^_partial_<0.10 |
|  | QL | **F_2,44_=16.3; P<0.001; η^2^_partial_=0.43** | F_1,22_=0.9; P=0.596; η^2^_partial_<0.10 | F_2,44_=1.1; P=0.315; η^2^_partial_<0.10 |
| L4/L5 | LM | **F_1.2,26.8_=30.6; P<0.001; η^2^_partial_=0.58** | F_1,22_=0.5; P=0.489; η^2^_partial_<0.10 | F_1.2,26.8_=0.5; P=0.525; η^2^_partial_<0.10 |
|  | LES | F_2,44_=2.3; P=0.117; η^2^_partial_<0.10 | F_1,22_=0.1; P=0.864; η^2^_partial_<0.10 | F_2,44_=1.7; P=0.193; η^2^_partial_<0.10 |
|  | PM | F_2,44_=2.9; P=0.071; η^2^_partial_=0.12 | F_1,22_=2.1; P=0.165; η^2^_partial_<0.10 | F_2,44_=0.9; P=0.416; η^2^_partial_<0.10 |
|  | QL | - | - | - |
| L5/S1 | LM | **F_1.2,27.3_=28.7; P<0.001; η^2^_partial_=0.57** | F_1,22_=0.3; P=0.609; η^2^_partial_<0.10 | F_1.2,27.3_=0.1; P=0.822; η^2^_partial_<0.10 |
|  | LES | **F_1.6,34.5_=6.1; P=0.009; η^2^_partial_=0.22** | F_1,22_=0.8; P=0.372; η^2^_partial_<0.10 | F_1.6,34.5_=0.2; P=0.804; η^2^_partial_<0.10 |
|  | PM | F_2,44_=0.9; P=0.919; η^2^_partial_<0.10 | F_1,22_=1.4; P=0.246; η^2^_partial_<0.10 | F_2,44_=0.6; P=0.561; η^2^_partial_<0.10 |
|  | QL | - | - | - |

**Supplementary table 2.** Mean (± standard deviation) paraspinal muscle fat content of the whole muscle (%) from SR (N=12) and SR+FRED (N=12) at BDC2, HDT59, and R13.

| Muscle | Group | Time | Intervertebral disc level | | | | |  |
| --- | --- | --- | --- | --- | --- | --- | --- | --- |
|  |  |  | L1/L2 | L2/L3 | L3/L4 | L4/L5 | L5/S1 | |
| Lumbar multifidus | SR | BDC | 18.1±8.2 | 16.0±7.6 | 18.8±7.8 | 23.7±10.4 | 29.0±10.8 | |
|  |  | HDT59 | 20.2±9.0 | 18.5±7.8* | 21.9±8.5* | 27.8±10.8* | 34.1±11.4* | |
|  |  | R13 | 19.7±9.0 | 16.2±6.8† | 20.0±8.1† | 24.8±9.9*† | 29.6±10.7† | |
|  | SR+FRED | BDC | 13.1±3.8 | 11.9±4.3 | 13.5±5.3 | 16.9±7.3 | 20.6±6.1 | |
|  |  | HDT59 | 13.2±4.3 | 13.3±4.8* | 16.1±5.8* | 20.9±7.5* | 25.5±6.2* | |
|  |  | R13 | 13.2±3.7 | 11.5±3.5† | 13.7±5.1† | 17.9±6.8*† | 21.4±6.3† | |
| Lumbar erector spinae | SR | BDC | 11.0±4.0 | 12.5±4.6 | 14.7±5.9 | 23.2±11.3 | 41.0±13.6 | |
|  |  | HDT59 | 12.7±4.5* | 15.1±5.8* | 17.4±7.3* | 25.8±12.5* | 43.5±14.2* | |
|  |  | R13 | 10.8±4.0† | 13.3±5.1† | 15.8±6.6† | 25.1±12.6 | 40.2±14.7† | |
|  | SR+FRED | BDC | 8.1±1.9 | 9.6±2.8 | 11.1±3.3 | 16.0±3.5 | 29.6±6.7 | |
|  |  | HDT59 | 9.4±2.6* | 10.9±2.8* | 13.0±3.7* | 17.8±4.8* | 32.3±6.1* | |
|  |  | R13 | 8.4±1.9† | 9.6±2.2† | 11.6±3.2† | 16.1±4.7 | 28.9±4.9† | |
| Psoas major | SR | BDC | 9.2±2.6 | 10.2±2.4 | 10.1±2.5 | 10.2±2.2 | 9.8±2.2 | |
|  |  | HDT59 | 9.2±2.6 | 10.4±2.5 | 9.9±2.2 | 10.5±2.2 | 10.6±2.1 | |
|  |  | R13 | 9.3±3.0 | 10.2±2.7 | 10.6±2.7 | 10.3±2.3 | 10.2±1.9 | |
|  | SR+FRED | BDC | 8.8±1.6 | 9.5±1.0 | 9.5±1.2 | 10.2±1.5 | 10.2±1.6 | |
|  |  | HDT59 | 7.9±1.6 | 8.9±1.3 | 9.7±1.6 | 10.6±1.5 | 10.6±2.2 | |
|  |  | R13 | 10.2±4.4 | 9.0±1.2 | 9.4±1.6 | 9.6±1.1 | 9.1±1.3 | |
| Quadratus lumborum | SR | BDC | 9.1±3.0 | 9.2±2.7 | 8.4±2.1 | - | - | |
|  |  | HDT59 | 8.6±2.7 | 9.1±2.6 | 8.1±2.0 | - | - | |
|  |  | R13 | 8.2±2.7 | 8.7±2.7 | 8.9±2.5 | - | - | |
|  | SR+FRED | BDC | 8.0±1.8 | 7.9±1.2 | 7.5±1.4 | - | - | |
|  |  | HDT59 | 7.7±2.1 | 7.9±1.2 | 6.9±1.1 | - | - | |
|  |  | R13 | 8.3±2.7 | 7.4±1.5 | 7.4±2.0 | - | - | |

* Pairwise comparisons (Bonferroni adjusted) relative to baseline (BDC) value (P < 0.05).

† Pairwise comparisons (Bonferroni adjusted) relative to the end of head-down tilt (HDT59) bed rest value (P < 0.05).

SR - Standard Reconditioning; SR+FRED - Standard Reconditioning supplemented with Functional Re-adaptive Exercise Device; BDC – Baseline data collection; HDT – Head-down tilt; R – Recovery.

**Supplementary table 3.** Results of the 2-way mixed model ANOVA of muscle paraspinal muscle fat content (whole muscle) volume using Time (BDC2, HDT59, and R13) as within factors, and Group (SR and SR+FRED) as between-group factor. Boldface indicated P < 0.05.

| Intervertebral  disc level | Muscle volume | 2-way mixed model ANOVA | | |
| --- | --- | --- | --- | --- |
|  |  | Time | Group | Time*Group |
| L1/L2 | LM | F_2,44_=2.4; P=0.103; η^2^_partial_=0.10 | **F_1,22_=5.1; P=0.033; η^2^_partial_=0.19** | F_2,44_=1.8; P=0.184; η^2^_partial_<0.10 |
|  | LES | **F_2,44_=26.7; P<0.001; η^2^_partial_=0.55** | **F_1,22_=4.8; P=0.040; η^2^_partial_=0.18** | F_2,44_=1.5; P=0.223; η^2^_partial_<0.10 |
|  | PM | F_2,44_=2.1; P=0.162; η^2^_partial_<0.10 | F_1,22_=0.7; P=0.789; η^2^_partial_<0.10 | F_2,44_=1.9; P=0.162; η^2^_partial<_0.10 |
|  | QL | F_2,44_=0.5; P=0.623; η^2^_partial_<0.10 | F_1,22_=0.1; P=0.710; η^2^_partial_<0.10 | F_2,44_=1.8; P=0.200; η^2^_partial_<0.10 |
| L2/L3 | LM | **F_2,44_=17.7; P<0.001; η^2^_partial_=0.45** | F_1,22_=3.7; P=0.067; η^2^_partial_=0.15 | F_2,44_=0.81; P=0.451; η^2^_partial_<0.10 |
|  | LES | **F_2,44_=40.2; P<0.001; η^2^_partial_=0.65** | **F_1,22_=4.7; P=0.041; η^2^_partial_=0.18** | F_2,44_=3.7; P=0.060; η^2^_partial=_0.15 |
|  | PM | F_2,44_=0.7; P=0500; η^2^_partial_<0.10 | F_1,22_=2.1; P=0.166; η^2^_partial_<0.10 | F_2,44_=1.7; P=0.193; η^2^_partial_<0.10 |
|  | QL | F_2,44_=1.6; P=0.207; η^2^_partial_<0.10 | F_1,22_=2.6; P=0.120; η^2^_partial_=0.10 | F_2,44_=0.4; P=0.959; η^2^_partial_<0.10 |
| L3/L4 | LM | **F_2,44_=44.6; P<0.001; η^2^_partial_=0.67** | **F_1,22_=4.4; P=0.049; η^2^_partial_=0.17** | F_2,44_=1.4; P=0.263; η^2^_partial_<0.10 |
|  | LES | **F_2,44_=29.9; P<0.001; η^2^_partial_=0.58** | F_1,22_=3.7; P=0.068; η^2^_partial_=0.14 | F_2,44_=1.1; P=0.331; η^2^_partial_<0.10 |
|  | PM | F_2,44_=0.5; P=0.596; η^2^_partial_<0.10 | F_1,22_=0.8; P=0.385; η^2^_partial_<0.10 | F_2,44_=2.2; P=0.128; η^2^_partial_<0.10 |
|  | QL | F_2,44_=2.2; P=0.124; η^2^_partial_<0.10 | F_1,22_=3.0; P=0.095; η^2^_partial_<0.10 | F_2,44_=0.5; P=0.630; η^2^_partial_<0.10 |
| L4/L5 | LM | **F_2,44_=51.0; P<0.001; η^2^_partial_=0.70** | F_1,22_=3.6; P=0.070; η^2^_partial_=0.14 | F_2,44_=0.1; P=0.986; η^2^_partial_<0.10 |
|  | LES | **F_2,44_=7.6; P=0.001; η^2^_partial_=0.26** | **F_1,22_=4.8; P=0.038; η^2^_partial_=0.18** | F_2,44_=1.4; P=0.266; η^2^_partial_<0.10 |
|  | PM | F_2,44_=3.0; P=0.058; η^2^_partial_=0.12 | F_1,22_=0.4; P=0.717; η^2^_partial_<0.10 | F_2,44_=2.0; P=0.151; η^2^_partial_<0.10 |
|  | QL | - | - | - |
| L5/S1 | LM | **F_2,44_=79.4; P<0.001; η^2^_partial_=0.78** | **F_1,22_=5.4; P=0.029; η^2^_partial_=0.20** | F_2,44_=0.1; P=0.888; η^2^_partial_<0.10 |
|  | LES | **F_2,44_=15.3; P<0.001; η^2^_partial_=0.41** | **F_1,22_=6.6; P=0.017; η^2^_partial_=0.23** | F_2,44_=0.1; P=0.992; η^2^_partial_<0.10 |
|  | PM | F_2,44_=2.9; P=0.070; η^2^_partial_=0.11 | F_1,22_=0.1; P=0.699; η^2^_partial_<0.10 | F_2,44_=1.7; P=0.187; η^2^_partial_<0.10 |
|  | QL | - | - | - |

**Supplementary table 4.** Mean (± standard deviation) of lumbar multifidus from SR (N=12) and SR+FRED (N=12), and results of the 3-way mixed model ANOVA of paraspinal muscle fat concent (%) using Time (BDC2, HDT59, and R13) and Quartile (Q1, Q2, Q3 and Q4) as within factors, and Group (SR and SR+FRED) as between-group factor. Q1 = medial; Q4 = lateral. Boldface indicated P < 0.05.

| Lumbar multifidus | | | | | | | | | | | | | | | | |
| --- | --- | --- | --- | --- | --- | --- | --- | --- | --- | --- | --- | --- | --- | --- | --- | --- |
| Intervertebral  disc level | Quartile | | SR | | | | | | | SR+FRED | | | | | | |
|  |  |  | BDC2 | | | HDT59 | | R13 | | BDC2 | | HDT59 | | R13 | | |
| L1/L2 | Q1 | | 23.3±12.0 | | | 26.1±9.1 | | 24.7±13.7 | | 18.7±6.2 | | 20.4±6.4 | | 18.2±5.5 | | |
|  | Q2 | | 15.5±9.1 | | | 17.5±9.3 | | 14.8±4.0 | | 11.2±4.4 | | 11.1±3.6 | | 12.0±4.0 | | |
|  | Q3 | | 12.9±5.7 | | | 14.6±6.7 | | 14.7±7.0 | | 9.1±2.6 | | 9.5±3.2 | | 9.8±2.7 | | |
|  | Q4 | | 17.2±8.1 | | | 19.8±9.7 | | 18.5±8.4 | | 10.1±2.6 | | 11.2±4.1 | | 11.2±2.7 | | |
| L2/L3 | Q1 | | 24.3±12.1 | | | 27.1±11.6 | | 24.9±12.2 | | 18.1±7.9 | | 20.4±8.7 | | 17.8±7.6 | | |
|  | Q2 | | 14.0±7.5 | | | 16.5±8.6 | | 14.5±7.6 | | 9.9±4.0 | | 11.4±4.4 | | 9.9±3.0 | | |
|  | Q3 | | 10.4±4.6 | | | 12.6±5.5 | | 10.9±4.3 | | 8.4±2.9 | | 9.3±3.3 | | 8.5±2.4 | | |
|  | Q4 | | 15.1±6.9 | | | 17.1±7.6 | | 13.5±3.8 | | 10.8±5.5 | | 11.8±4.3 | | 9.8±2.8 | | |
| L3/L4 | Q1 | | 28.6±11.0 | | | 32.2±11.8 | | 30.7±12.2 | | 22.0±8.8 | | 25.4±9.0 | | 22.2±8.7 | | |
|  | Q2 | | 17.4±9.5 | | | 20.1±10.3 | | 17.5±9.4 | | 11.8±5.5 | | 14.3±6.2 | | 12.3±5.4 | | |
|  | Q3 | | 10.9±5.6 | | | 13.1±6.5 | | 11.5±5.6 | | 8.3±3.0 | | 9.7±3.4 | | 8.4±3.1 | | |
|  | Q4 | | 17.4±6.8 | | | 21.3±8.7 | | 19.4±8.8 | | 11.4±6.4 | | 14.2±5.7 | | 11.5±3.9 | | |
| L4/L5 | Q1 | | 33.4±11.3 | | | 37.9±11.4 | | 35.7±12.1 | | 27.2±12.3 | | 31.3±11.5 | | 28.8±11.6 | | |
|  | Q2 | | 22.2±9.6 | | | 27.1±11.5 | | 25.0±10.8 | | 15.7±7.3 | | 19.2±8.0 | | 17.0±7.3 | | |
|  | Q3 | | 14.6±7.1 | | | 16.4±8.3 | | 15.6±7.4 | | 9.6±4.5 | | 12.3±4.8 | | 10.0±4.3 | | |
|  | Q4 | | 24.1±14.4 | | | 28.9±13.7 | | 22.4±11.2 | | 14.6±5.8 | | 20.3±7.1 | | 15.2±11.6 | | |
| L5/S1 | Q1 | | 34.9±11.2 | | | 40.0±11.9 | | 38.2±11.9 | | 25.8±10.2 | | 31.1±9.6 | | 27.0±10.4 | | |
|  | Q2 | | 29.1±10.2 | | | 33.7±11.0 | | 31.0±11.1 | | 21.3±7.6 | | 24.1±7.6 | | 22.5±7.5 | | |
|  | Q3 | | 21.5±9.8 | | | 25.8±10.1 | | 22.2±9.9 | | 15.2±4.6 | | 17.7±4.0 | | 16.2±5.2 | | |
|  | Q4 | | 29.7±14.1 | | | 35.6±15.2 | | 26.3±12.4 | | 19.9±6.0 | | 27.9±8.8 | | 19.5±6.0 | | |
| 3-way mixed model ANOVA | | | | | | | | | | | | | | | |  |
| Intervertebral  disc level | | Time | | Quartile | Group | | Time*  Quartile | | Time*  Group | | Quartile*  Group | | Time*  Quartile*  Group | |  |  |
| L1/L2 | | **F_2,44_=5.0**  **P=0.011**  **η^2^_partial_=0.18** | | **F_1.4,30.7_=32.0**  **P<0.001**  **η^2^_partial_=0.60** | **F_1,22_=4.5**  **P=0.045**  **η^2^_partial_=0.17** | | F_3.5,77.8_=1.9  P=0.118  η^2^_partial_<0.10 | | F_2,44_=1.7  P=0.193  η^2^_partial_<0.10 | | F_1.4,93.9_=0.8  P=0.428  η^2^_partial_<0.10 | | F_3.6,38.0_=2.2  P=0.087  η^2^_partial_<0.10 | |  |  |
| L2/L3 | | **F_2,44_=18.1**  **P<0.001**  **η^2^_partial_=0.45** | | **F_1.3,28.5_=42.8**  **P<0.001**  **η^2^_partial_=0.66** | F_1,22_=3.8  P=0.064  η^2^_partial_=0.15 | | F_3.1,67.5_=1.9  P=0.142  η^2^_partial_<0.10 | | F_2,44_=0.9  P=0.422  η^2^_partial_<0.10 | | F_1.3,28.6_=1.1  P=0.360  η^2^_partial_<0.10 | | F_3.1,67.5_=0.4  P=0.750  η^2^_partial_<0.10 | |  |  |
| L3/L4 | | **F_2,44_=45.5**  **P<0.001**  **η^2^_partial_=0.67** | | **F_1.8,40.6_=69.1**  **P<0.001**  **η^2^_partial_=0.76** | **F_1,22_=4.4**  **P=0.047**  **η^2^_partial_=0.17** | | F_3.5,78.2_=1.2  P=0.294  η^2^_partial_<0.10 | | F_2,44_=1.4  P=0.264  η^2^_partial_<0.10 | | F_1.8.40.6_=1.4  P=0.276  η^2^_partial_<0.10 | | F_3.5,78.2_=0.7  P=0.538  η^2^_partial_<0.10 | |  |  |
| L4/L5 | | **F_2,44_=49.3**  **P<0.001**  **η^2^_partial_=0.69** | | **F_2.2,47.7_=80.8**  **P<0.001**  **η^2^_partial_=0.79** | F_1,22_=3.7  P=0.066  η^2^_partial_=0.15 | | **F_2.4,51.2_=7.6**  **P<0.001**  **η^2^_partial_=0.26** | | F_2,44_=0.1  P=0.988  η^2^_partial_<0.10 | | F_2.2,47.8_=0.7  P=0.544  η^2^_partial_<0.10 | | F_3.0,66.2_=1.5  P=0.200  η^2^_partial_<0.10 | |  |  |
| L5/S1 | | **F_2,44_=78.3**  **P<0.001**  **η^2^_partial_=0.78** | | **F_1.9,42.9_=30.0**  **P<0.001**  **η^2^_partial_=0.58** | **F_1,22_=5.5**  **P=0.029**  **η^2^_partial_=0.20** | | **F_3.4,73.4_=12.5**  **P<0.001**  **η^2^_partial_=0.36** | | F_2,44_=0.2  P=0.820  η^2^_partial_<0.10 | | F_1.9,42.9_=0.4  P=0.671  η^2^_partial_<0.10 | | F_3.3,73.4_=2.1  P=0.099  η^2^_partial_<0.10 | |  |  |

**Supplementary table 5**. Mean (± standard deviation) of lumbar erector spinae from SR (N=12) and SR+FRED (N=12), and results of the 3-way mixed model ANOVA of paraspinal muscle fat content (%) using Time (BDC2, HDT59, and R13) and Quartile (Q1, Q2, Q3, and Q4) as within factors, and Group (SR and SR+FRED) as between-group factor. Q1 = medial; Q4 = lateral. Boldface indicated P < 0.05.

| Lumbar erector spinae | | | | | | | | | | | | | | |
| --- | --- | --- | --- | --- | --- | --- | --- | --- | --- | --- | --- | --- | --- | --- |
| Intervertebral  disc level | Quartile | SR | | | | | | | SR+FRED | | | | | |
|  |  | BDC2 | | HDT59 | | | R13 | | BDC2 | | HDT59 | | R13 | |
| L1/L2 | Q1 | 13.2±4.6 | | 15.0±5.3 | | | 12.1±4.0 | | 8.6±2.1 | | 10.7±3.2 | | 10.1±2.7 | |
|  | Q2 | 11.4±4.7 | | 14.2±5.9 | | | 11.3±4.8 | | 7.7±2.6 | | 9.6±3.7 | | 8.3±2.6 | |
|  | Q3 | 10.1±3.4 | | 11.8±4.7 | | | 10.3±4.0 | | 8.0±1.8 | | 9.1±2.2 | | 7.9±1.9 | |
|  | Q4 | 9.6±4.0 | | 10.6±4.0 | | | 9.7±3.7 | | 7.8±2.0 | | 8.7±2.9 | | 7.8±2.4 | |
| L2/L3 | Q1 | 15.5±6.2 | | 17.9±6.6 | | | 16.3±7.0 | | 10.5±3.6 | | 11.9±3.8 | | 10.1±6.2 | |
|  | Q2 | 14.4±5.8 | | 18.4±7.8 | | | 15.4±6.7 | | 11.3±3.0 | | 13.0±4.8 | | 11.7±3.8 | |
|  | Q3 | 10.2±3.5 | | 12.6±4.5 | | | 11.2±3.8 | | 9.0±2.2 | | 10.4±2.4 | | 9.1±1.7 | |
|  | Q4 | 10.2±4.8 | | 11.7±5.8 | | | 10.5±4.8 | | 7.9±2.5 | | 8.6±2.2 | | 7.7±2.0 | |
| L3/L4 | Q1 | 22.1±9.1 | | 25.3±11.2 | | | 24.5±11.6 | | 15.2±4.1 | | 16.9±5.1 | | 15.1±4.7 | |
|  | Q2 | 14.3±6.3 | | 17.2±7.7 | | | 15.5±6.6 | | 11.6±4.6 | | 14.3±4.8 | | 12.7±4.2 | |
|  | Q3 | 11.1±5.0 | | 13.3±6.4 | | | 11.3±4.8 | | 8.6±3.6 | | 10.2±4.2 | | 9.3±4.0 | |
|  | Q4 | 11.2±3.9 | | 13.5±5.0 | | | 11.7±4.5 | | 9.3±2.5 | | 10.7±3.1 | | 9.3±2.2 | |
| L4/L5 | Q1 | 38.5±17.4 | | 41.6±19.6 | | | 40.6±19.3 | | 25.8±6.0 | | 27.0±7.3 | | 25.4±7.8 | |
|  | Q2 | 18.5±8.9 | | 21.5±10.0 | | | 20.4±10.3 | | 13.8±3.6 | | 16.2±4.5 | | 13.7±4.8 | |
|  | Q3 | 16.0±9.1 | | 18.2±10.2 | | | 17.9±10.5 | | 11.9±2.9 | | 13.5±4.4 | | 11.8±3.6 | |
|  | Q4 | 17.9±9.8 | | 20.0±10.2 | | | 19.5±10.5 | | 12.2±3.9 | | 14.1±5.7 | | 12.8±5.3 | |
| L5/S1 | Q1 | 53.6±15.9 | | 56.1±15.0 | | | 54.8±15.3 | | 41.3±8.9 | | 46.3±7.3 | | 44.4±6.6 | |
|  | Q2 | 33.5±16.7 | | 34.6±17.8 | | | 34.3±18.9 | | 18.4±4.7 | | 20.8±5.0 | | 19.5±15.5 | |
|  | Q3 | 33.4±12.5 | | 35.7±14.2 | | | 31.7±13.6 | | 24.0±12.5 | | 25.4±8.0 | | 21.7±5.8 | |
|  | Q4 | 41.1±11.3 | | 45.3±13.2 | | | 38.0±12.9 | | 32.9±12.0 | | 34.4±11.3 | | 28.7±8.7 | |
| 3-way mixed model ANOVA | | | | | | | | | | | | | |  |
| Intervertebral  disc level | Time | | Quartile | | Group | Time*  Quartile | | Time*  Group | | Quartile*  Group | | Time*  Quartile*  Group | |  |
| L1/L2 | **F_2,44_=29.7**  **P<0.001**  **η^2^_partial_=0.57** | | **F_2.3,52.7_=10.9**  **P<0.001**  **η^2^_partial_=0.33** | | **F_1,22_=5.1**  **P=0.034**  **η^2^_partial_=0.19** | F_3.7,81.6_=2.0  P=0.118  η^2^_partial_=0.10 | | F_2,44_=2.3  P=0.113  η^2^_partial_=0.10 | | F_2.4,52.7_=1.7  P=0.178  η^2^_partial_<0.10 | | F_3.7,81.6_=2.2  P=0.083  η^2^_partial_=0.10 | |  |
| L2/L3 | **F_2,44_=40.2**  **P<0.001**  **η^2^_partial_=0.65** | | **F_2.2,48.9_=20.8**  **P<0.001**  **η^2^_partial_=0.49** | | **F_1,22_=4.8**  **P=0.039**  **η^2^_partial_=0.18** | F_2.7,59.6_=2.8  P=0.053  η^2^_partial_=0.13 | | F_2,44_=2.5  P=0.095  η^2^_partial_=0.10 | | F_2.2,48.9_=2.7  P=0.071  η^2^_partial_=0.10 | | F_2.7,59.6_=1.6  P=0.15  η^2^_partial_<0.10 | |  |
| L3/L4 | **F_2,44_=30.4**  **P<0.001**  **η^2^_partial_=0.58** | | **F_1.9,42.4_=47.7**  **P<0.001**  **η^2^_partial_=0.68** | | F_1,22_=3.6  P=0.071  η^2^_partial_=0.14 | F_3.0,65.7_=0.9  P=0.460  η^2^_partial_<0.10 | | F_2,44_=1.1  P=0.328  η^2^_partial_<0.10 | | F_1.9.42.4_=1.9  P=0.176  η^2^_partial_<0.10 | | F_3.0,65.7_=1.5  P=0.229  η^2^_partial_<0.10 | |  |
| L4/L5 | **F_2,44_=7.3**  **P=0.002**  **η^2^_partial_=0.25** | | **F_1.5,33.6_=81.2**  **P<0.001**  **η^2^_partial_=0.79** | | **F_1,22_=4.7**  **P=0.040**  **η^2^_partial_=0.18** | F_3.7,82.1_=0.6  P=0.730  η^2^_partial_<0.10 | | F_2,44_=1.3  P=0.271  η^2^_partial_<0.10 | | F_1.5,33.6_=1.5  P=0.235  η^2^_partial_<0.10 | | F_3.0,66.2_=0.6  P=0.649  η^2^_partial_<0.10 | |  |
| L5/S1 | **F_1.4,30.8_=15.1**  **P<0.001**  **η^2^_partial_=0.41** | | **F_2.0,44.5_=80.7**  **P<0.001**  **η^2^_partial_=0.79** | | **F_1,22_=6.7**  **P=0.016**  **η^2^_partial_=0.24** | **F_2.7,60.2_=6.3**  **P=0.001**  **η^2^_partial_=0.22** | | F_1.4,30.8_=0.1  P=0.994  η^2^_partial_<0.10 | | F_2.0,44.5_=1.0  P=0.374  η^2^_partial_<0.10 | | F_2.7,60.2_=0.9  P=0.426  η^2^_partial_<0.10 | |  |

**Supplementary table 6**. Mean (± standard deviation) of psoas major from SR (N=12) and SR+FRED (N=12), and results of the 3-way mixed model ANOVA of paraspinal muscle fat content (%) using Time (BDC2, HDT59, and R13) and Quartile (Q1, Q2, Q3, and Q4) as within factors, and Group (SR and SR+FRED) as between-group factor. Q1 = medial; Q4 = lateral. Boldface indicated P < 0.05.

| Psoas major | | | | | | | | | | | | | | |  |
| --- | --- | --- | --- | --- | --- | --- | --- | --- | --- | --- | --- | --- | --- | --- | --- |
| Intervertebral  disc level | Quartile | | SR | | | | | | SR+FRED | | | | | |  |
|  |  |  | BDC2 | | HDT59 | | R13 | | BDC2 | | HDT59 | | R13 | |  |
| L1/L2 | Q1 | | 13.3±4.5 | | 14.0±5.0 | | 14.4±6.6 | | 14.2±4.0 | | 12.4±3.8 | | 16.0±8.2 | |  |
|  | Q2 | | 8.4±2.6 | | 7.9±2.1 | | 8.4±2.7 | | 7.7±0.9 | | 7.2±1.4 | | 9.6±4.7 | |  |
|  | Q3 | | 8.2±2.5 | | 8.0±2.4 | | 7.9±3.1 | | 7.4±1.1 | | 6.7±1.0 | | 8.6±3.1 | |  |
|  | Q4 | | 8.0±2.3 | | 8.2±2.9 | | 7.4±2.0 | | 7.5±2.1 | | 6.7±1.5 | | 8.0±2.9 | |  |
| L2/L3 | Q1 | | 15.0±4.6 | | 15.5±2.4 | | 16.4±5.9 | | 14.1±2.7 | | 12.5±3.1 | | 13.5±2.9 | |  |
|  | Q2 | | 8.8±2.4 | | 8.4±2.0 | | 8.5±2.4 | | 7.8±0.9 | | 7.3±1.0 | | 7.8±1.1 | |  |
|  | Q3 | | 8.4±1.7 | | 8.3±1.8 | | 7.9±1.9 | | 7.9±0.8 | | 7.4±1.1 | | 7.5±0.7 | |  |
|  | Q4 | | 8.6±1.8 | | 8.6±2.1 | | 7.9±1.5 | | 8.3±1.0 | | 7.6±1.3 | | 7.4±1.1 | |  |
| L3/L4 | Q1 | | 13.6±3.8 | | 13.5±3.8 | | 15.1±5.9 | | 12.7±2.9 | | 13.1±4.0 | | 13.0±3.9 | |  |
|  | Q2 | | 9.4±2.9 | | 9.2±2.4 | | 9.8±2.5 | | 8.5±1.0 | | 8.7±1.3 | | 8.5±1.0 | |  |
|  | Q3 | | 8.3±2.2 | | 7.9±1.5 | | 8.4±1.6 | | 8.0±1.1 | | 8.0±1.0 | | 7.9±1.0 | |  |
|  | Q4 | | 9.0±2.0 | | 9.0±2.1 | | 9.1±2.1 | | 8.7±1.1 | | 8.9±1.1 | | 8.2±0.9 | |  |
| L4/L5 | Q1 | | 13.5±4.3 | | 13.2±3.4 | | 13.4±4.4 | | 12.3±2.9 | | 14.0±3.6 | | 12.3±2.5 | |  |
|  | Q2 | | 9.5±2.0 | | 9.9±2.0 | | 9.7±2.2 | | 9.8±1.7 | | 10.0±1.7 | | 9.3±1.5 | |  |
|  | Q3 | | 8.5±1.7 | | 8.8±1.1 | | 8.7±2.0 | | 8.8±1.1 | | 8.9±1.1 | | 8.1±1.1 | |  |
|  | Q4 | | 9.5±2.1 | | 9.9±2.2 | | 9.5±1.8 | | 9.9±1.3 | | 9.9±1.5 | | 8.7±1.7 | |  |
| L5/S1 | Q1 | | 9.9±2.0 | | 10.8±2.7 | | 10.1±2.2 | | 10.9±2.3 | | 11.4±3.0 | | 10.1±2.8 | |  |
|  | Q2 | | 9.2±2.0 | | 9.9±2.0 | | 9.8±1.9 | | 9.5±1.9 | | 9.4±2.1 | | 8.5±1.4 | |  |
|  | Q3 | | 9.8±2.8 | | 10.4±2.4 | | 10.3±2.2 | | 9.7±1.8 | | 10.4±2.4 | | 8.8±1.7 | |  |
|  | Q4 | | 10.3±2.8 | | 11.3±2.2 | | 10.6±2.6 | | 10.6±1.9 | | 11.4±2.6 | | 9.2±1.9 | |  |
| 3-way mixed model ANOVA | | | | | | | | | | | | | | | |
| Intervertebral  disc level | | Time | | Quartile | | Group | | Time*  Quartile | | Time*  Group | | Quartile*  Group | | Time*  Quartile*  Group | |
| L1/L2 | | F_2,44_=2.1  P=0.139  η^2^_partial_<0.10 | | **F_1.4,31.4_=60.4**  **P<0.001**  **η^2^_partial_=0.73** | | F_1,22_=0.1  P=0.872  η^2^_partial_<0.10 | | F_2.3,51.4_=1.4  P=0.273  η^2^_partial_<0.10 | | F_2,44_=2.1  P=0.131  η^2^_partial_<0.10 | | F_1.4,31.4_=0.2  P=0.737  η^2^_partial_<0.10 | | F_2.3,51.4_=0.6  P=0.574  η^2^_partial_<0.10 | |
| L2/L3 | | F_2,44_=1.7  P=0.200  η^2^_partial_<0.10 | | **F_1.1,24.7_=97.8**  **P<0.001**  **η^2^_partial_=0.82** | | F_1,22_=2.1  P=0.158  η^2^_partial_<0.10 | | F_3.1,67.7_=2.7  P=0.057  η^2^_partial_=0.11 | | F_2,44_=1.7  P=0.188  η^2^_partial_<0.10 | | F_1.1,24.7_=1.5  P=0.232  η^2^_partial_<0.10 | | F_3.1,67.7_=1.8  P=0.145  η^2^_partial_<0.10 | |
| L3/L4 | | F_2,44_=0.5  P=0.629  η^2^_partial_<0.19 | | **F_1.4,30.1_=53.1**  **P<0.001**  **η^2^_partial_=0.71** | | F_1,22_=0.8  P=0.391  η^2^_partial_<0.10 | | F_2.4,53.7_=2.0  P=0.132  η^2^_partial_<0.10 | | F_2,44_=2.0  P=0.152  η^2^_partial_=<0.10 | | F_1.4.30.1_=0.4  P=0.576  η^2^_partial_<0.10 | | F_2.4,53.7_=0.4  P=0.724  η^2^_partial_<0.10 | |
| L4/L5 | | F_2,44_=3.0  P=0.058  η^2^_partial_=0.10 | | **F_1.4,30.1_=40.1**  **P<0.001**  **η^2^_partial_=0.65** | | F_1,22_=0.1  P=0.745  η^2^_partial_<0.10 | | F_4.0,74.7_=0.5  P=0.726  η^2^_partial_<0.10 | | F_2,44_=1.9  P=0.173  η^2^_partial_<0.10 | | F_1.4,30.1_=0.3  P=0.828  η^2^_partial_<0.10 | | F_4.0,74.7_=1.2  P=0.332  η^2^_partial_<0.10 | |
| L5/S1 | | F_2,44_=2.8  P=0.072  η^2^_partial_=0.10 | | **F_3,66_=5.8**  **P=0.001**  **η^2^_partial_=0.23** | | F_1,22_=0.1  P=0.724  η^2^_partial_<0.10 | | F_6,132_=1.1  P=0.355  η^2^_partial_<0.10 | | F_2,44_=1.7  P=0.187  η^2^_partial_<0.10 | | F_3,66_=1.1  P=0.354  η^2^_partial_<0.10 | | F_6,132_=0.6  P=0.718  η^2^_partial_<0.10 | |

**Supplementary table 7.** Mean (± standard deviation) of quadratus lumborum from SR (N=12) and SR+FRED (N=12), and results of the 3-way mixed model ANOVA of paraspinal muscle fat content (%) using Time (BDC2, HDT59, and R13) and Quartile (Q1, Q2, Q3, and Q4) as within factors, and Group (SR and SR+FRED) as between-group factor. Q1 = medial; Q4 = lateral. Boldface indicated P < 0.05.

| Quadratus lumborum | | | | | | | | | | | | |  |
| --- | --- | --- | --- | --- | --- | --- | --- | --- | --- | --- | --- | --- | --- |
| Intervertebral  disc level | Quartile | | SR | | | | SR+FRED | | | | | |  |
|  |  |  | BDC2 | HDT59 | R13 | | BDC2 | | HDT59 | | R13 | |  |
| L1/L2 | Q1 | | 10.5±4.4 | 11.1±4.6 | 10.8±4.7 | | 9.4±3.8 | | 8.9±2.5 | | 10.6±3.6 | |  |
|  | Q2 | | 8.3±2.6 | 8.5±3.2 | 7.8±3.2 | | 6.9±1.8 | | 7.3±2.1 | | 7.5±3.1 | |  |
|  | Q3 | | 7.5±2.2 | 6.9±2.5 | 7.2±2.7 | | 6.6±1.8 | | 7.0±2.5 | | 7.2±2.7 | |  |
|  | Q4 | | 10.3±4.4 | 8.9±3.5 | 7.8±2.1 | | 9.1±2.9 | | 8.2±3.4 | | 8.7±3.8 | |  |
| L2/L3 | Q1 | | 12.9±5.1 | 13.3±5.1 | 13.4±5.4 | | 10.8±2.7 | | 11.0±2.7 | | 10.6±3.2 | |  |
|  | Q2 | | 8.5±3.1 | 7.7±3.0 | 8.1±2.5 | | 6.7±1.1 | | 6.9±1.3 | | 6.9±1.9 | |  |
|  | Q3 | | 6.7±1.5 | 6.7±1.7 | 6.3±1.7 | | 6.2±0.6 | | 6.1±1.4 | | 6.0±1.2 | |  |
|  | Q4 | | 8.7±2.3 | 8.5±2.2 | 6.9±2.2 | | 8.0±2.0 | | 7.7±1.6 | | 6.0±1.6 | |  |
| L3/L4 | Q1 | | 10.4±3.1 | 10.3±2.9 | 11.7±4.1 | | 9.2±2.6 | | 8.3±1.3 | | 8.9±2.7 | |  |
|  | Q2 | | 7.5±2.3 | 7.3±1.9 | 7.6±2.1 | | 6.3±1.2 | | 6.2±1.2 | | 6.5±1.9 | |  |
|  | Q3 | | 6.6±2.2 | 6.7±2.3 | 7.0±1.7 | | 6.0±1.0 | | 6.0±1.2 | | 6.4±2.0 | |  |
|  | Q4 | | 9.0±1.9 | 8.2±2.1 | 9.2±4.0 | | 8.4±2.4 | | 7.3±1.7 | | 7.9±2.2 | |  |
| 3-way mixed model ANOVA | | | | | | | | | | | | | |
| Intervertebral  disc level | | Time | Quartile | Group | | Time*  Quartile | | Time*  Group | | Quartile*  Group | | Time*  Quartile*  Group | |
| L1/L2 | | F_2,44_=0.2  P=0.795  η^2^_partial_<0.10 | **F_1.5,33.5_=12.7**  **P<0.001**  **η^2^_partial_=0.37** | F_1,22_=0.8  P=0.386  η^2^_partial_<0.10 | | F_3.4,75.0_=1.3  P=0.291  η^2^_partial_<0.10 | | F_2,44_=0.9  P=0.425  η^2^_partial_<0.10 | | F_1.5,33.5_=0.6  P=+.489  η^2^_partial_<0.10 | | F_3.4,75.0_=0.8  P=0.540  η^2^_partial_<0.10 | |
| L2/L3 | | F_2,44_=1.6  P=0.220  η^2^_partial_<0.10 | **F_1.3,28.9_=54.0**  **P<0.001**  **η^2^_partial_=0.71** | F_1,22_=2.6  P=0.121  η^2^_partial_=0.11 | | F_3.4,74.9_=0.7  P=0.728  η^2^_partial_<0.10 | | F_2,44_=0.1  P=0.968  η^2^_partial_<0.10 | | F_1.3,28.9_=1.6  P=0.222  η^2^_partial_<0.10 | | F_3.4,74.9_=0.5  P=0.728  η^2^_partial_<0.10 | |
| L3/L4 | | F_1.5,34.6_=2.2  P=0.136  η^2^_partial_<0.10 | **F_1.6,35.8_=41.5**  **P<0.001**  **η^2^_partial_=0.65** | F_1,22_=3.0  P=0.097  η^2^_partial_=0.12 | | F_3.5,76.7_=1.0  P=0.365  η^2^_partial_<0.10 | | F_1.5,34.6_=0.4  P=0.62  η^2^_partial_<0.10 | | F_1.6.35.8_=1.6  P=0.219  η^2^_partial_<0.10 | | F_3.5,76.7_=0.8  P=0.531  η^2^_partial_<0.10 | |
